# A Rare Dual Complication of First-Line Anti-tubercular Therapy: A Case Report of Drug Reaction With Eosinophilia and Systemic Symptoms (DRESS) Syndrome and Ethambutol-Induced Optic Neuropathy

**DOI:** 10.7759/cureus.115207

**Published:** 2026-08-26

**Authors:** Koustav Kundu, Aouf Alazzawi

**Affiliations:** 1 Acute and General Internal Medicine, Bedford Hospital, Bedfordshire Hospitals NHS Foundation Trust, Bedford, GBR

**Keywords:** anti-tubercular therapy (att), drug hypersensitivity reactions, drug reaction with eosinophilia and systemic symptoms (dress) syndrome, ethambutol-induced optic neuropathy (eon), tuberculosis

## Abstract

Drug reaction with eosinophilia and systemic symptoms (DRESS) syndrome is an extremely rare and serious systemic drug reaction, often posing significant diagnostic challenge to clinicians. Ethambutol-induced optic neuropathy (EON) is a well-recognised but often insidious complication of first-line anti-tubercular therapy (ATT), typically manifesting as reduced visual acuity, and visual field defects. We report the case of a 78-year-old woman with confirmed diagnosis of pulmonary tuberculosis, who simultaneously developed probable DRESS syndrome, secondary to rifampicin and EON shortly after initiation of the standard first-line ATT regimen (isoniazid, rifampicin, pyrazinamide, and ethambutol). Early diagnosis and prompt withdrawal of the offending drugs helped her to achieve complete recovery with no complications. This case highlights the importance of a high index of suspicion and early recognition of drug-induced adverse reactions in patients on multi-drug ATT regimens, as timely diagnosis and management can help in preventing serious complications like permanent vision loss, and also death.

## Introduction

Tuberculosis (TB) remains a global public health emergency, with an estimated 10.7 million new cases and 1.08 million deaths recorded in 2024 [1]. The standard first-line anti-tubercular therapy (ATT) regimen comprises isoniazid (H), rifampicin (R), pyrazinamide (Z), and ethambutol (E), commonly referred to as the HRZE regimen, and is the cornerstone of TB treatment worldwide [1]. While this regimen is highly effective, each of its constituent drugs carries a recognised adverse effect profile, and clinicians must remain vigilant for drug-induced reactions that can complicate treatment.

Drug reaction with eosinophilia and systemic symptoms (DRESS) syndrome is a severe, potentially life-threatening drug hypersensitivity reaction characterised by an extensive morbilliform skin rash, fever, lymphadenopathy, haematological abnormalities and visceral organ involvement [2]. Its incidence is estimated to range from one in every 1,000 to one in every 10,000 drug exposures [2]. It typically manifests two to eight weeks after initiation of the offending agent and carries a reported mortality of up to 10%, most commonly from fulminant hepatitis with hepatic necrosis [2]. According to Gell and Coombs' classification of hypersensitivity, the pathogenesis involves a delayed T-cell-mediated hypersensitivity reaction (Type IVb) characterized by eosinophilic inflammation [2]. Among patients receiving first-line ATT, rifampicin and isoniazid are the most frequently implicated causative drugs [3,4]. Diagnosis is standardised using the European Registry of Severe Cutaneous Adverse Reactions (RegiSCAR) scoring system, which classifies cases as possible, probable, or definite based on clinical and laboratory criteria [5].

Ethambutol-induced optic neuropathy (EON) classically presents with painless, bilateral visual loss, red-green colour vision deficiency, and central or centrocaecal scotoma [6,7]. The prevalence of EON from a standard dose of ethambutol has been reported to be 0.7-1.29% [6]. The pathophysiology behind EON remains incompletely understood. The most common proposed mechanism involves mitochondrial dysfunction in the retinal ganglion cells, which leads to cellular stress and apoptosis of optic nerve fibres. Risk factors include higher cumulative doses, prolonged duration of therapy, advanced age, systemic diseases, malnutrition, and pre-existing renal or hepatic dysfunction [8]. Early identification and prompt discontinuation of ethambutol remains the single most important intervention to maximise the likelihood of visual recovery, which again may be partial or complete [6,7].

Although both DRESS syndrome and EON are individually recognised complications of first-line ATT, no prior reports of these two serious adverse drug reactions occurring simultaneously in a single patient on HRZE therapy have been identified in the literature. We present this case to highlight the diagnostic complexity and therapeutic dilemmas posed by this dual complication, and to illustrate the importance of close clinical and laboratory monitoring with early specialist involvement for patient safety and favourable outcomes.

## Case presentation

A 78-year-old woman presented to the Emergency Department with a two- to three-day history of fever, blurred vision, extreme fatigue, and reduced mobility due to bilateral ankle swelling. She had been commenced on standard first-line ATT (HRZE) 19 days prior, following a confirmed diagnosis of pulmonary TB. Her past medical history was significant for rheumatoid arthritis, ulcerative colitis, adult-onset asthma, osteoporosis, and anaemia of chronic disease.

On initial assessment, she was pyrexial with a temperature of 38.5°C. Both lower limbs were erythematous, oedematous, and tender. The initial differential diagnoses included cellulitis, deep vein thrombosis, and drug-induced hypersensitivity reaction, given the recent commencement of ATT. Ultrasound Doppler ruled out deep vein thrombosis. Empirical antibiotics were commenced for suspected cellulitis pending further evaluation. Blood investigations revealed eosinophilia of 0.68 × 10⁹/L, hyponatremia of 125 mmol/L and deranged renal parameters, with a serum urea of 10.8 mmol/L and serum creatinine of 157 µmol/L, consistent with acute kidney injury (AKI) Stage 1. Other labs were grossly normal, including liver function tests (Table 1).

**Table 1:** Admission blood investigations * Denotes abnormal values outside reference range

Parameter	Patient Value	Unit	Reference Range
Bone Profile			
Albumin	*28	g/L	35-50
Calcium	2.45	mmol/L	2.2-2.6
Adjusted Calcium	*2.69	mmol/L	2.2-2.6
Alkaline Phosphatase (ALP)	*28	U/L	30-130
Phosphate	*1.53	mmol/L	0.8-1.5
Full Blood Count			
White Cell Count	10.3	10⁹/L	4-11
Haemoglobin	130	g/L	120-160
Platelets	427	10⁹/L	150-400
Mean Platelet Volume (MPV)	*7.3	fL	7.8-11
RBC	4.4	10¹²/L	4-5.2
Haematocrit	0.38	L/L	0.36 – 0.46
Mean Corpuscular Volume (MCV)	88	fL	80 – 100
Mean Cell Haemoglobin (MCH)	29.8	pg	27 – 32
Mean Corpuscular Hemoglobin Concentration (MCHC)	337	g/L	280 – 355
Red Cell Distribution Width (RDW)	13.4	%	11.8 – 14.8
Neutrophils	*8.47	10⁹/L	2-7
Lymphocytes	*0.62	10⁹/L	1-3
Monocytes	0.5	10⁹/L	0.2 – 1
Eosinophils	*0.68	10⁹/L	0 – 0.4
Basophils	0.03	10⁹/L	0.02 – 0.1
Nucleated Red Blood Cell (NRBC)	<0.5	10⁹/L	0 – 0.5
Liver Function			
Total Protein	63	g/L	60 – 80
Calculated Globulin	35	g/L	-
Total Bilirubin	10	μmol/L	0 – 20
Alanine Aminotransferase (ALT)	15	U/L	0 – 32
Renal Function			
Sodium	*125	mmol/L	133 – 146
Potassium	4.8	mmol/L	3.5 – 5.3
Creatinine	*157	μmol/L	44 – 80
Others			
Urea	*10.8	mmol/L	2.5 – 7.8
Acute Kidney Injury (AKI) Stage	*1	—	0

**Figure 1:**
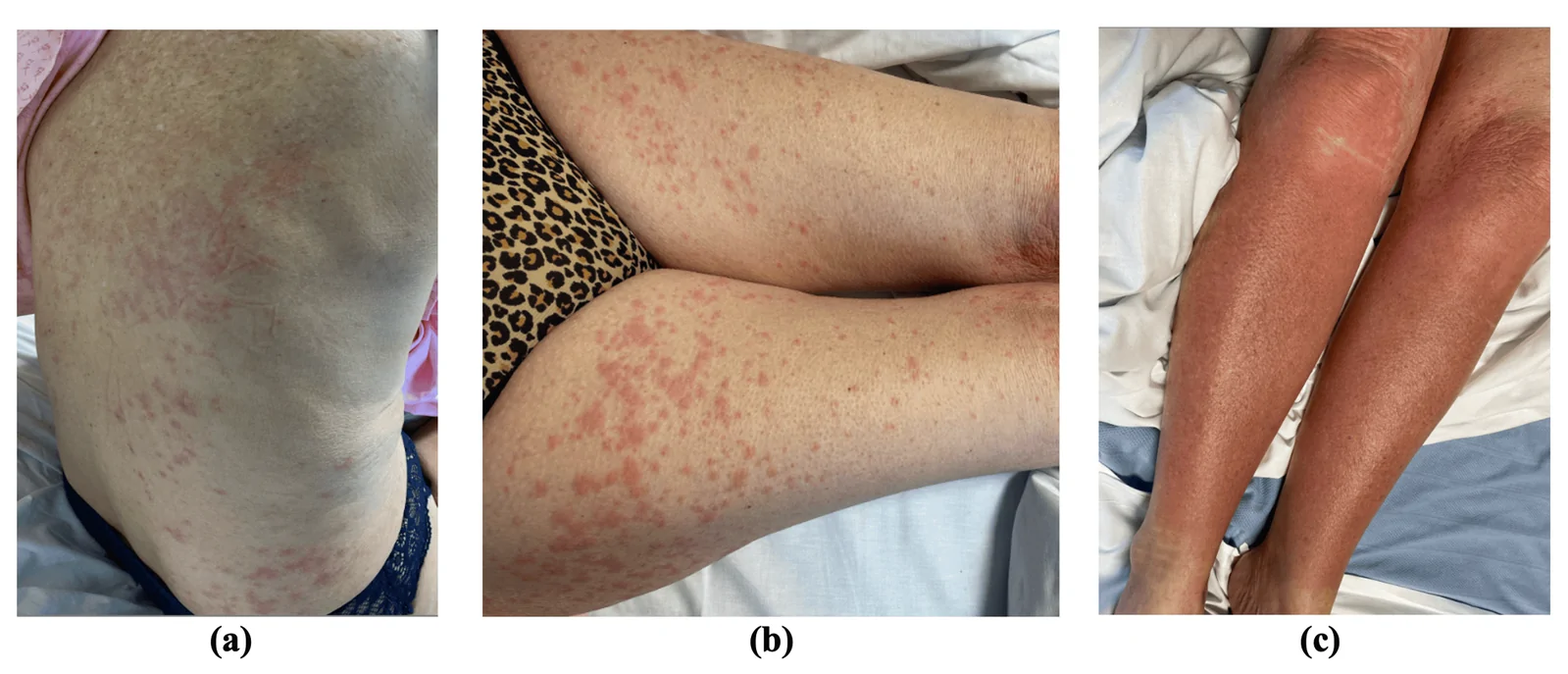
Rash covering more than 50% of body surface area Sites: (a) Back (b) Bilateral Thighs (c) Bilateral Legs

On Day 3 of admission, she developed a sudden-onset, widespread morbilliform rash over the abdomen, back, and lower limbs (Figure 1), covering more than 50% of body surface. Upon Dermatology review, she was commenced on an antihistamine and topical steroid. 

While fever, bilateral ankle swelling, and renal impairment were initially nonspecific, the combination of eosinophilia, widespread morbilliform rash, and renal involvement temporally associated with ATT commencement raised concerns for drug hypersensitivity reaction. Alternative diagnoses like Stevens-Johnson syndrome and toxic epidermal necrolysis were excluded on the basis of the morbilliform rather than blistering or erosive nature of the rash, and the absence of mucosal involvement. A viral exanthema was considered unlikely given the temporal relationship with ATT commencement, the presence of eosinophilia, and the absence of prodromal viral symptoms or positive serology.

Ophthalmological examination revealed marked reduction in visual acuity, loss of colour vision and bilateral visual field defects. The patient's visual acuity and colour vision findings are summarised in Table 2.

**Table 2:** Visual acuity and colour vision results * Denotes abnormal results beyond the reference range

Category	Investigation	Right Eye	Left Eye	Normal Reference
Visual Acuity	Best Corrected Visual Acuity (LogMAR / Snellen)	*0.42 (6/15)	*0.42 (6/15)	0.00 (6/6)
Colour Vision	Ishihara Colour Vision Test (13 plates total)	*1/13 plates correct	*2/13 plates correct	13/13 plates correct

**Figure 2:**
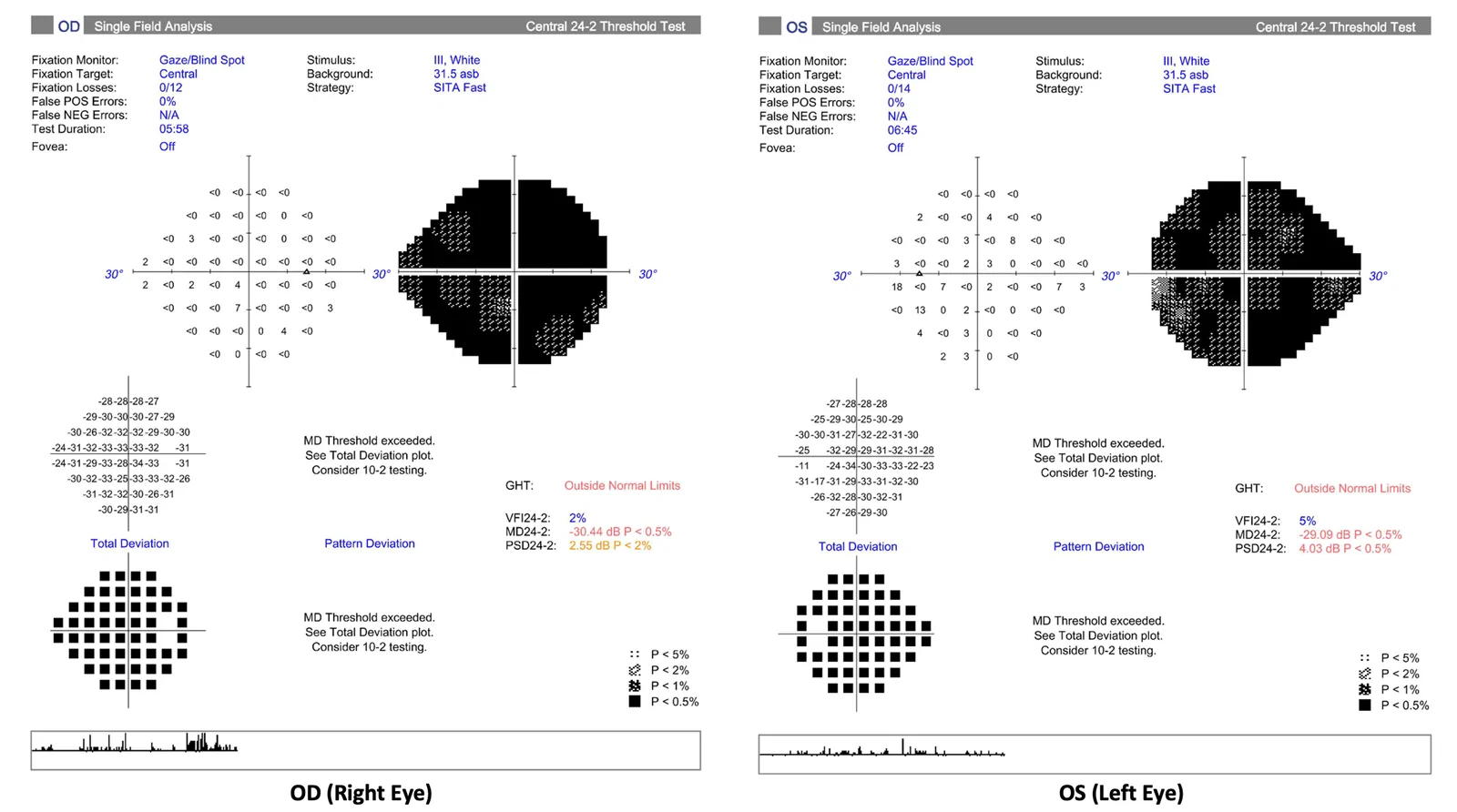
Humphrey visual field testing, demonstrating bilateral scotoma with near-complete vision loss OD: *oculus dexter* (right eye); OS: *oculus sinister* (left eye); MD: mean deviation; VFI: visual field index; GHT: Glaucoma Hemifield Test; dB: decibels

Humphrey Visual Field analysis (Humphrey Field Analyzer; Zeiss, Oberkochen, Baden-Württemberg, Germany)using the Central 24-2 threshold test and SITA FAST (Swedish Interactive Threshold Algorithm Fast) strategy revealed near-complete bilateral visual field loss. Mean deviation (MD) was severely depressed at −30.44 dB (P < 0.5%) in the right eye, and −29.09 dB (P < 0.5%) in the left eye, with the MD threshold exceeded in both eyes. The visual field index was critically reduced at 2% in the right eye and 5% in the left eye, against a normal value of 100%. The Glaucoma Hemifield Test was outside normal limits bilaterally. Fixation losses were 0/12 in the right eye and 0/14 in the left eye, and false positive errors were 0% in both eyes, confirming the reliability of the test results. The visual field maps demonstrated dense bilateral scotoma with near-complete field loss, consistent with involvement of the papillomacular bundle, the characteristic anatomical target of ethambutol toxicity (Figure 2). 

Alternative causes of bilateral optic neuropathy like inflammatory optic neuritis, ischaemic optic neuropathy, and compressive lesions were considered and excluded. Clinically, there was no pain on eye movement, no optic disc swelling, and no features of demyelinating disease. CT head imaging was performed and showed no acute intracranial abnormality, excluding a compressive lesion as the cause of her visual field defects.

**Figure 3:**
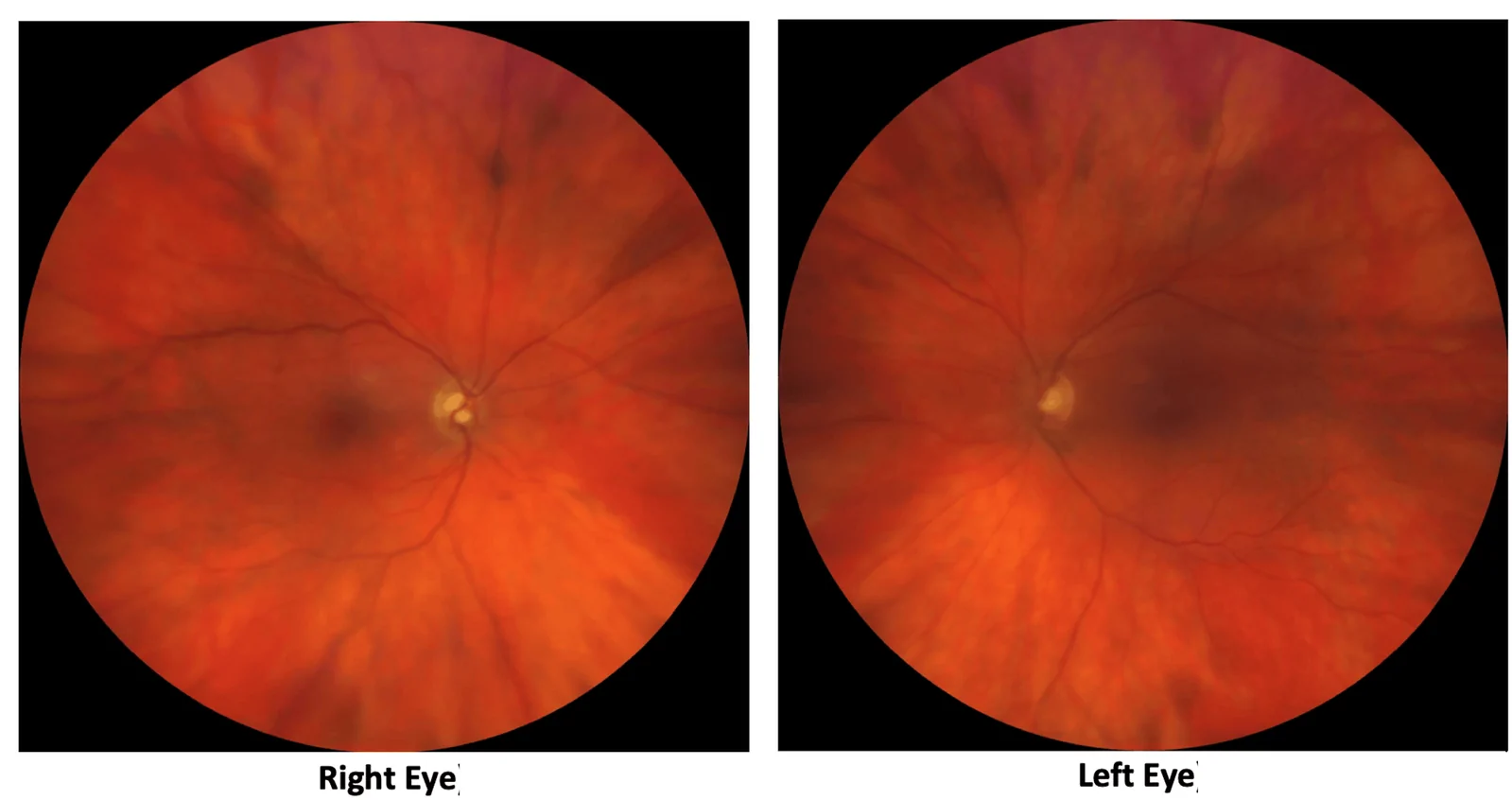
Fundoscopy Images, showing bilateral normal optic disc.

Colour fundus photography showed a normal optic disc appearance bilaterally with no pallor or swelling (Figure 3), consistent with early-stage ethambutol-induced optic neuropathy in which structural disc changes may not yet be apparent despite significant functional loss.

Specialist ophthalmological review supported a diagnosis of ethambutol-induced optic neuropathy, and ethambutol was permanently discontinued. A multidisciplinary decision was made to temporarily suspend all ATT for one week from Day 5 to Day 11 of admission, with a planned sequential re-challenge thereafter, reintroducing each drug individually in the order of isoniazid, rifampicin, and pyrazinamide, with close monitoring for clinical deterioration or drug-specific adverse effects.

**Figure 4:**
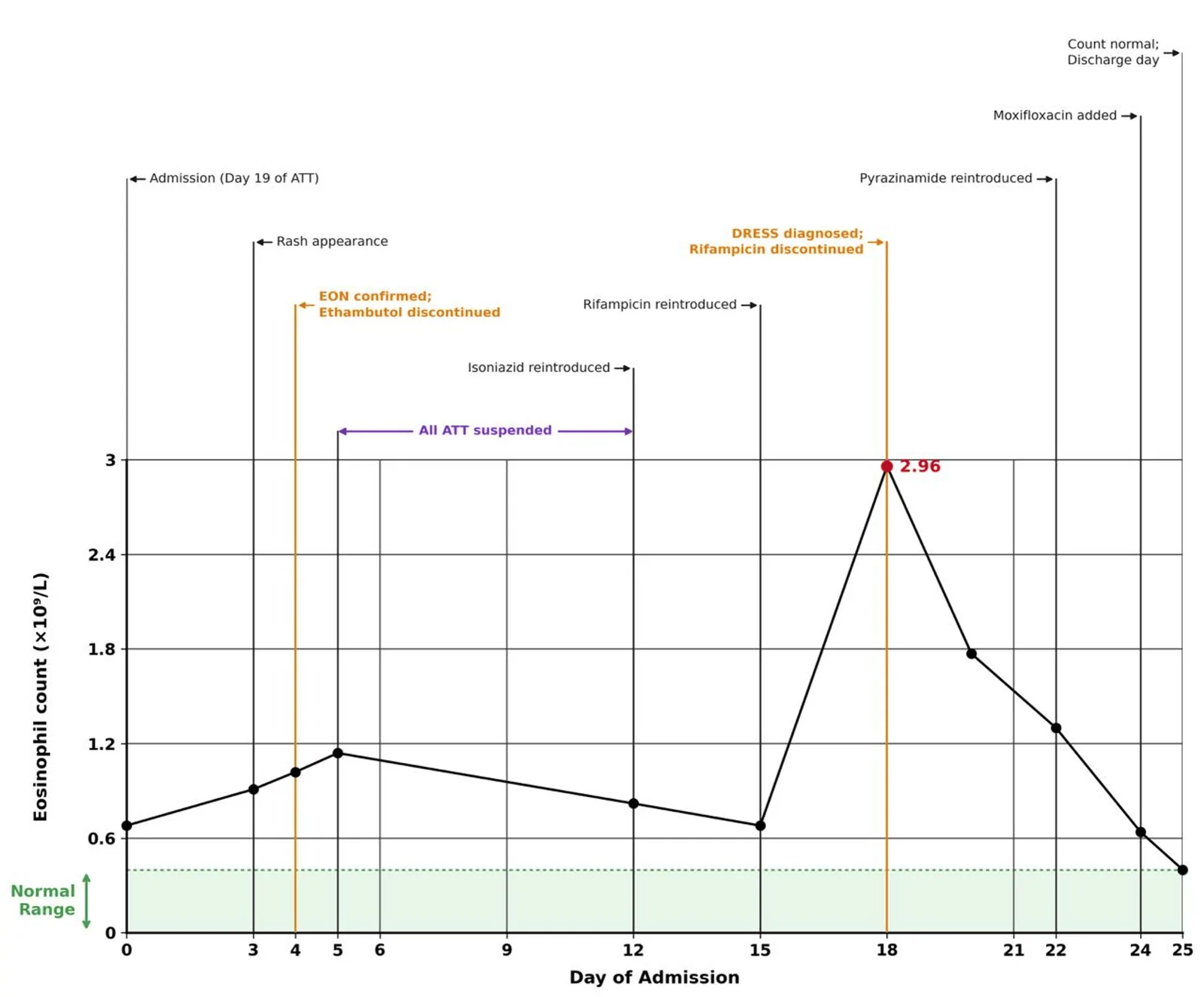
Serial eosinophil count and key clinical events throughout admission ATT: anti-tubercular therapy; EON: ethambutol-induced optic neuropathy; DRESS: drug reaction with eosinophilia and systemic symptoms.

Over the following week with ATT remaining paused, the patient showed clinical improvement; eosinophilia trended downward and the rash began to clear. Following the one-week suspension period, ATT was reintroduced as planned. Isoniazid was reintroduced on Day 12 of admission and tolerated without incident. However, upon reintroduction of rifampicin on Day 15, eosinophilia jumped to a peak of 2.96 × 10⁹/L on Day 18, a surge directly temporally related to rifampicin reintroduction (Figure 4).

At this stage, a probable diagnosis of DRESS syndrome secondary to rifampicin (RegiSCAR score 5) was made (Table 4). The RegiSCAR scoring system (Table 3) was applied to objectively confirm this diagnosis [5], and the patient-specific application is detailed in Table 4. 

**Table 3:** Standard RegiSCAR Scoring for DRESS Diagnosis Interpretation: Score 2–3: possible DRESS; Score 4–5: probable DRESS; Score > 5: definite DRESS; Reference: Kardaun et al. [5]. ANA, antinuclear antibody; HAV, hepatitis A virus; HBV, hepatitis B virus; HCV, hepatitis C virus; DRESS: drug reaction with eosinophilia and systemic symptoms; RegiSCAR: European Registry of Severe Cutaneous Adverse Reactions

Criterion	Finding/Category	Score
Systemic and Haematological Features		
Fever ≥ 38.5°C	No/Unknown	-1
	Yes	0
Enlarged lymph nodes (≥ 2 sites, > 1 cm)	No/Unknown	0
	Yes	1
Atypical lymphocytes	No/Unknown	0
	Yes	1
Eosinophilia	< 0.7 × 10⁹/L or < 10% (no eosinophilia)	0
	0.7–1.49 × 10⁹/L or 10–19.9%	1
	≥ 1.5 × 10⁹/L or ≥ 20%	2
Skin Involvement		
Skin rash extent > 50% Body Surface Area	No/Unknown	0
	Yes	1
Skin rash morphology (≥ 2 of: oedema, infiltration, purpura, scaling)	No	-1
	Unknown	0
	Yes	1
Biopsy suggesting DRESS	No	-1
	Yes/Unknown	0
Organ Involvement		
Internal organ(s) involved (Liver, Kidney, Lung, Muscle/heart, Pancreas, Others)	0 organs	0
	1 organ	1
	≥ 2 organs	2
Resolution and Exclusion of Alternatives		
Resolution in ≥ 15 days	No/Unknown	-1
	Yes	0
Alternative diagnoses excluded (ANA, HIV, Blood culture, HAV, HBV, HCV, Mycoplasma, Chlamydia)	Unknown/Any positive/ <3 negative	0
	None positive and ≥ 3 negative	1
Total RegiSCAR Score (Possible Range)		−4 to +9

**Table 4:** Patient Assessment on RegiSCAR Scoring *Denotes positive findings in patient; Score 5 : Probable DRESS Syndrome (Reference : Score < 2: No case; 2–3: Possible; 4–5: Probable; > 5: Definite). DRESS: drug reaction with eosinophilia and systemic symptoms; RegiSCAR: European Registry of Severe Cutaneous Adverse Reactions

RegiSCAR Criterion		Findings in This Patient	Points Available	Points Scored
Fever ≥ 38.5 °C		*Present (38.5 °C on admission)	−1 (absent); 0 (present)	0
Enlarged lymph nodes		Absent	0 (absent) ; +1 (present)	0
Eosinophilia		*Peak eosinophilia 2.96 × 10⁹/L on rifampicin re-challenge	0 (absent); +1 (0.7–1.49 × 10⁹/L); +2 (≥ 1.5 × 10⁹/L)	2
Atypical lymphocytes		Absent	0 (absent); +1 (present)	0
Skin rash extent (% body surface area)		*Widespread rash covering > 50% body surface area	0 (≤ 50% or absent); +1 (> 50%)	1
Skin rash morphology		*Morbilliform erythematous rash with edema, infiltration, purpura and scaling	−1 (inconsistent or absent); 0 (unknown); +1 (consistent)	1
Biopsy suggesting DRESS		Clinical diagnosis made without biopsy	−1 (inconsistent); 0 (not done / consistent)	0
Organ involvement		*Kidney : Acute Kidney Injury (Elevated Urea and Creatinine); nil other organs affected	0 (absent); +1 (1 organ affected); +2 (≥ 2 organs affected)	1
Resolution ≥ 15 days		*Rash resolved over more than 15 days	−1 (< 15 days); 0 (≥ 15 days)	0
Exclusion of other potential causes		Full exclusion panel not performed.	0 (unknown); 1 (None positive and ≥ 3 tests negative)	0
Total RegiSCAR Score	-		Maximum: 9	5

Rifampicin was permanently discontinued on Day 18 and later substituted with moxifloxacin [9], as advised by the Respiratory team. Pyrazinamide was subsequently reintroduced and tolerated well. 

Over the following days, eosinophilia resolved to within the normal reference range, renal parameters returned to the patient's baseline, and the rash showed significant resolution. The patient reported symptomatic improvement, with no further visual disturbance or mobility difficulties. Systemic corticosteroid was not required at any stage. She was discharged on Day 25 on a modified ATT regimen comprising isoniazid, moxifloxacin, and pyrazinamide as advised by the Respiratory team, with complete resolution of rash and bilateral lower limb swelling at the time of discharge.

At two-week follow-up, the patient reported feeling well, with a return to her baseline and normal daily activities. There was no recurrence of rash. At three-month follow-up at the eye clinic, best corrected visual acuity had returned to 6/6 bilaterally, and Ishihara colour vision testing was normal at 13/13 plates correct in both eyes, representing complete recovery from the presenting deficits. No persistent visual field defects were identified.

The timeline of key clinical events and drug adjustments throughout admission is summarised in Table 5.

**Table 5:** Timeline of key clinical events throughout admission ATT: anti-tubercular therapy; DRESS: drug reaction with eosinophilia and systemic symtoms

Day	Event
Pre-admission	ATT (isoniazid, rifampicin, pyrazinamide, ethambutol) started
Day 0	Admission in hospital - Day 19 of ATT
Day 3	Rash appearance
Day 4	Ethambutol-induced optic neuropathy confirmed; Ethambutol discontinued
Day 5	All ATT suspended
Day 12	Isoniazid reintroduced
Day 15	Rifampicin reintroduced
Day 18	Eosinophils peak; DRESS diagnosed (RegiSCAR 5); Rifampicin discontinued
Day 22	Pyrazinamide reintroduced
Day 24	Moxifloxacin added
Day 25	Eosinophils normal; symptoms improved; rash resolved; discharged
2 weeks post-discharge	Clinically well with no rash recurrence
3 months post-discharge	Visual acuity fully restored

## Discussion

This case presents a rare dual drug-induced complication arising from first-line ATT, DRESS syndrome secondary to rifampicin and EON secondary to ethambutol, occurring concurrently in an elderly patient with significant medical comorbidities. 

DRESS syndrome in the context of ATT

The hallmark features of DRESS syndrome typically emerge two to eight weeks following exposure to the offending agent, consistent with the timeline observed in our patient [2]. DRESS syndrome associated with ATT has been documented in the literature, with rifampicin and isoniazid being most frequently implicated [3,4]. In a retrospective cohort study of anti-tubercular drug-induced DRESS, rifampicin was identified as the causative drug in 84.2% of cases [10]. In our case, rifampicin was identified as the offending drug. Causality was strongly supported by a positive dechallenge and rechallenge; eosinophilia and rash improved during ATT suspension, but eosinophilia recurred specifically upon rifampicin reintroduction while isoniazid and pyrazinamide were tolerated without incident. This sequential rechallenge pattern is considered strong evidence of causation, as it isolates the offending agent from co-administered drugs sharing a similar timeline. The RegiSCAR scoring system yielded a score of 5, consistent with a probable diagnosis [5]. Sequential re-challenge, rather than empirical substitution of the entire regimen, enabled precise identification of the offending drug while preserving essential anti-tubercular agents. This approach is consistent with published experience by Morán-Mariños et al., who demonstrated the feasibility and safety of structured desensitisation and re-desensitisation protocols to recover anti-tubercular drugs in patients with DRESS syndrome [10].

Ethambutol-induced optic neuropathy

EON is a dose- and duration-dependent toxic adverse effect of ethambutol, affecting the papillomacular bundle of the optic nerve [6]. While onset typically manifests after two to eight months of ethambutol therapy, it can occur considerably earlier in patients with multiple predisposing risk factors. Kim et al. identified advanced age, female sex, higher cumulative dose, renal or liver disease, systemic diseases (hypertension, diabetes, hyperlipidaemia) and nutritional disorders as independent risk factors for EON in a large cohort study of 204,598 ethambutol users [8]. Our patient carried several of these risk factors, including advanced age, female sex, renal impairment and anaemia of chronic disease. These likely compounded her susceptibility to ethambutol toxicity through impaired drug clearance and made her symptoms arise within the first month, which is atypical in nature. Causality was supported by a consistent temporal relationship and improvement following withdrawal (positive dechallenge), though formal rechallenge was deliberately not attempted given the risk of irreversible visual loss. The pathophysiological mechanisms underlying EON remain incompletely understood, and to date no targeted treatment or preventative strategy has been established [6]. 

In our patient, the early involvement of the Ophthalmology team and timely diagnosis with early discontinuation of ethambutol were probably the reasons behind complete restoration of visual acuity within three months. Published data suggest that only approximately 30-50% of patients achieve full visual recovery, with many experiencing persistent deficits despite prompt drug cessation [7]. The complete recovery observed in our patient is therefore a favourable outcome, likely attributable to the early stage of toxicity at the time of ethambutol discontinuation.

Diagnostic and therapeutic complexity

The simultaneous occurrence of DRESS syndrome and EON in this patient created a particularly challenging clinical scenario, requiring a multidisciplinary approach. Early involvement of Dermatology, Ophthalmology, and Respiratory Medicine was essential to guide appropriate drug withdrawal, facilitate sequential re-challenge where indicated, and tailor alternative regimens to maintain adequate anti-tubercular efficacy.

This case also illustrates the compounding risk factors that predispose elderly patients to ATT-related toxicity. Advanced age is independently associated with both DRESS syndrome and EON, and the presence of multiple comorbidities, including rheumatoid arthritis, ulcerative colitis, renal impairment, and anaemia of chronic disease, further complicated the clinical picture and heightened vulnerability to adverse drug reactions [8]. These factors highlight the importance of pre-treatment baseline assessment, including renal function, visual acuity, colour vision testing, and careful counselling regarding potential adverse effects, prior to commencing ATT in elderly or multimorbid patients. 

Importantly, our patient achieved complete clinical recovery, resolution of rash, normalisation of eosinophil count and renal parameters, and restoration of visual acuity, without the need for systemic corticosteroids. This stands in contrast to more severe cases of ATT-associated DRESS in which systemic corticosteroids were typically required [3,4]; and re-emphasises that, when DRESS syndrome is identified early, and the offending drug is promptly withdrawn, disease progression may be averted without escalation to immunosuppressive therapy.

Implications for clinical practice

This case advocates for a structured approach to monitoring patients commenced on ATT, particularly those who are elderly and multimorbid. We suggest that baseline and interval assessments should include a full blood count with differential (to detect eosinophilia), renal and hepatic function tests, and formal ophthalmological evaluation incorporating visual acuity and colour vision testing prior to and during ethambutol therapy. Patients and their caregivers should be educated about the early symptoms of DRESS syndrome (new rash, fever, oedema) and visual toxicity (blurred vision, colour vision changes) to facilitate timely medical review.

In a nutshell, this case carries several important learning points for clinicians managing patients on ATT: (i) A high index of suspicion for drug-induced adverse reactions must be maintained throughout the course of ATT, particularly in elderly and multimorbid patients who carry a heightened vulnerability to toxic and hypersensitivity reactions, (ii) Pre-treatment baseline assessment, including renal function, full blood count, and formal ophthalmological evaluation, is essential to stratify risk and facilitate early detection of emerging complications, (iii) Sequential drug re-challenge, rather than empirical substitution of the entire regimen, enables precise identification of the offending agent while preserving as many essential anti-tubercular drugs as possible, (iv) As no targeted treatment for EON currently exists, prompt ethambutol withdrawal upon earliest suspicion of visual toxicity remains the single most effective intervention to maximise the chances of visual recovery, and (v) Finally, this case reinforces that timely multidisciplinary collaboration is indispensable in navigating the competing diagnostic and therapeutic challenges posed by complex ATT-related adverse reactions, and can result in complete clinical recovery even in high-risk patients.

## Conclusions

We report a case of concurrent DRESS syndrome and EON in a patient receiving standard first-line ATT. Prompt recognition of both complications, supported by a structured sequential drug re-challenge protocol and early multidisciplinary involvement, was central to achieving a favourable outcome without the need for systemic corticosteroids. Permanent discontinuation of rifampicin and ethambutol necessitated modification of the ATT regimen, underscoring the importance of clinician familiarity with alternative agents and salvage regimens.

This case serves as a reminder that drug-induced adverse effects in the context of multi-drug ATT are not always singular. Elderly patients and those with significant comorbidities are at heightened risk of overlapping toxicities. A proactive monitoring approach, incorporating routine haematological, biochemical, and ophthalmological assessment from the outset of treatment, offers the best chance of early detection and a successful clinical outcome.
